# Proceedings of the Louisiana Medical Association

**Published:** 1878-04-20

**Authors:** 


### PROCEEDINGS OF THE LOUISIANA MEDICAL ASSOCIATION.

The Association was organized on the 4th of January last in the city of New Orleans. Officers elect for the year are as follows:

Dr. J. C. Egan, President.
Dr. S. M. Bemiss, First Vice President.
Dr. J. W. Dupree, Second Vice President.
Dr. G. A. B. Hayes, Third Vice President.
Dr. Thomas Layton, Recording Secretary.
Dr. S. S. Herrick, Corresponding Secretary.
Dr. G. K. Pratt, Treasurer.
Dr. S. E. Chailli, Orator.

The constitution presented by Dr. A. A. Lyon, the chairman of committee, is well gotten up, and the meeting was attended by a respectable number of intelligent and progressive medical gentlemen.

A committee from the association composed of Drs. Richardson, Watkins and Layton, addressed a circular to the physicians of the State upon the importance of establishing local medical societies, from which we extract the following valuable suggestions which we trust will be observed in Georgia and other States:

"1. By means of such organization the individual members thereof are stimulated to higher professional culture, and to closer and more systematic observation of the progress and treatment of disease.

"2. Each member's knowledge of prevailing diseases, of types of disease, and of the results of various methods of treatment in the same locality, is thus made the common property of all.

"3. The community of interest resulting from formal association, largely increases the influence of the profession upon general society, and thus serves as a principal means of diffusing information, and repressing empiricism.

"4. It is only through the formation and maintenance of numerous parish societies that a State Society may be made successful.

"5. Probably the most important consideration that we can mention, and one which we would most earnestly press upon your attention, is the fact that it is only by the formation of such parish or county societies, which shall act in concert with the State Society, that the profession can hope ever to accomplish anything whatever in the improvement of medical education, or in the inauguration and promotion of State Medicine, than which no subject of equal importance to the material welfare and happiness of the people can possibly be proposed to those who make and execute the laws of the Commonwealth.

"We would suggest that the organizations in those parishes in which the number of physicians is small, need not comprise a full complement of officers, a president and secretary being all that is necessary; nor is it essential that frequent meetings shall be held. In this way a society may be maintained at little or no expense, and without any serious tax upon the time of the members."

